# Correction: Synthesis of a furfural-based DOPO-containing co-curing agent for fire-safe epoxy resins

**DOI:** 10.1039/d0ra90016h

**Published:** 2020-02-25

**Authors:** Weiqi Xie, Shiwen Huang, Donglin Tang, Shumei Liu, Jianqing Zhao

**Affiliations:** School of Materials Science and Engineering, South China University of Technology Guangzhou 510640 P. R. China liusm@scut.edu.cn psjqzhao@scut.edu.cn +86-13611400566 +86-13609724000 +86-13611400566 +86-13609724000; Key Laboratory of Polymer Processing Engineering, Ministry of Education Guangzhou 510640 P. R. China

## Abstract

Correction for ‘Synthesis of a furfural-based DOPO-containing co-curing agent for fire-safe epoxy resins’ by Weiqi Xie *et al.*, *RSC Adv.*, 2020, **10**, 1956–1965.

The authors regret that an incorrect version of Fig. 8 was included in the original article. This was due to FTIR spectra of the commercial reference sample (DGEBA/DDM, EP-0) being confused with those of other samples. The correct version of Fig. 8 is presented below.

**Fig. 8:**
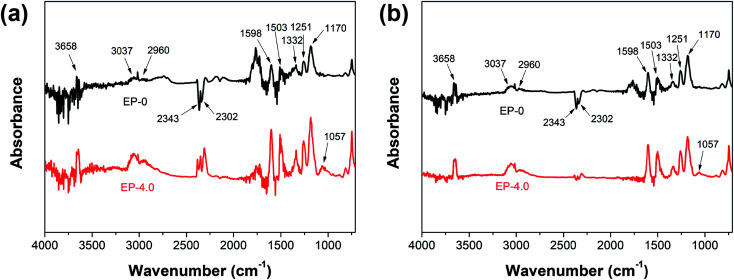
FTIR spectra of the pyrolysis products of EP-0 and EP-4.0 at (a) the initial and (b) maximum degradation temperatures.

The Royal Society of Chemistry apologises for these errors and any consequent inconvenience to authors and readers.

## Supplementary Material

